# Validation of an algorithm for sound-based voided volume estimation

**DOI:** 10.1038/s41598-023-50499-1

**Published:** 2024-01-02

**Authors:** Gyoohwan Jung, Hoyoung Ryu, Jeong Woo Lee, Seong Jin Jeong, Eric Margolis, Neel Grover, Sangchul Lee

**Affiliations:** 1 Department of Urology, Hanyang University College of Medicine, 222, Wangsimni-ro, Seongdong-gu, Seoul, Korea; 2 Department of Urology, Ewha Womans University College of Medicine, 52, Ewhayeodae-gil, Seodaemun-gu, Seoul, Korea; 3 Department of Urology, Kyung Hee University Medical Center, Kyung Hee University College of Medicine, 7-13, Kyungheedae-ro 6-gil, Dongdaemun-gu, Seoul, Korea; 4 Department of Urology, Seoul National University Bundang Hospital, 173-82, Gumi-ro, Bundang-gu, Seongnam-si, Gyeonggi-do Korea 13620; 5 Department of Urology, Seoul National University College of Medicine, 103 Daehakro, Seoul, 03080 Korea; 6 Hackensack Meridian School of Medicine, 340, Kingland St., Nutley, NJ 07110 USA; 7 New Jersey Urology, Englewood, NJ USA

**Keywords:** Urology, Software

## Abstract

A voiding diary is commonly used in clinical practice to monitor urinary tract health. However, manual recording and use of a measuring cup can cause significant inaccuracy and inconvenience. Recently sound-based voided volume estimation algorithms such as proudP have shown potential to accurately measure the voided volumes of patients urination while overcoming these inconveniences. In order to validate the sound-based voided volume estimation algorithm, we chose bodyweight change after urination as a reference value. Total 508 subjects from the United States and Korea were enrolled. 584 data points that have matching bodyweights change data and urination sound data were collected, and fivefold cross validation was performed in order to evaluate the model on all data in the dataset. The mean voided volume estimated by the algorithm was 202.6 mL (SD: ± 114.8) while the mean bodyweight change after urination was 208.0 g (SD: ± 121.5), and there was a strong linear correlation with high statistical significance (Pearson’s correlation coefficient = 0.92, p-value < 0.001). Two paired t-test showed the equivalence with bodyweight change data with 10 mL margin. Additionally, a Bland–Altman plot shows a mean difference of − 5.5 mL with LoA (− 98.0, 87.1). The results support high performance of the algorithm across the large population data from multi-site clinical trials.

## Introduction

Daily tracking of voiding parameters provides important information regarding patients’ urinary health^1^. In clinical practice, a voiding diary kept by patients is a useful tool recommended and utilized by physicians to assess a patient's urinary health^2^. These are typically measured at home by the patient who has to manually record the voided volume (VV) by reading the marking on a measuring cup^3^. The inconvenience of conducting multiple manual steps can contribute to poor compliance^4^. Additionally, there are high risks for inaccuracy caused by the lack of standard measuring cups and human mistakes occurred during manual reading and recording^5^.

In the past, several investigators attempted to demonstrate the performance of sound-based estimations that might solve those challenges^6–13^. Among those sound-based estimation algorithms, the proudP by Soundable Health, Inc (San Jose, CA, USA) is the only commercialized and the most active in clinical research.

However, it was found challenging to choose an appropriate standard measure because a commercial uroflowmeter usually requires subjects to urinate in a designated device while acoustic estimation analyzes the sound that the urine hits the water surface in a toilet bowl. Conventional ultrasound bladder volume scanner results^14^ or bodyweight changes before and after voiding^15^ were exploited as a standard reference. In fact, in clinical practice, urine weight has been commonly used to estimate VV. For example, gravimetric uroflowmeters, one of the commercially available types^16^, converts urine weight into volume. As a patient urinates into a specific beaker, a weight transducer in the uroflowmeter detects the change in receptacle weight and converts it into the VV^17^.

Therefore, in this study, we validate the algorithm for the VV estimation by comparing with the VV converted from bodyweight changes due to urination. The conversion was based on the assumption that urine specific gravity is 1. The error from the conversion would have little effect on the clinical practice^15^.

## Materials and methods

### Ethics statement

This study was approved by the local Institutional Review Board of Seoul National University Bundang Hospital (IRB No. B-2012-654-305) and Western Institutional Review Board Copernicus Group (IRB No. 20215311). All data used for analysis were anonymized. We obtained informed consent from all patients enrolled in the study. Personal identifiers were completely removed and the data were analyzed anonymously. All methods were performed in accordance with relevant guidelines and regulations.

### Study population and definitions

Subjects who were healthy volunteers or patients, aged over 18 years old, and able to provide informed consent to participate were eligible. Data collection occurred from September 27, 2021 to October 27, 2022 (Study 1), and from October 27, 2021 to July 14, 2022 (Study 2). Data were collected in the bathrooms at the hospital or clinic. Participants were allowed to provide multiple voiding sounds, and one void was registered as an independent event regardless of who provided it.

Exclusion criteria were retracted consents, the lack of matching data for either voiding sound or bodyweight change data, bodyweight change over the capacity of the sound-based algorithm (either under 10 g or over 1 kg). Additionally, recordings that failed to follow study instructions were excluded such as poor, incomplete, interrupted recording of voiding sound, voiding into another object that is not water in a toilet bowl, changes in conditions that can affect bodyweight such as consumption or excretion of food, or addition or removal of items carried by the subject.

### Procedure

Written informed consent was obtained from all enrolled subjects prior to data collection. Subjects were asked to complete a questionnaire with basic demographic questions including medical history. Right after measuring pre-void bodyweight, subjects recorded voiding sound using the iOS mobile application solely developed for data collection, which was immediately followed by post-void bodyweight measurement.

### Data collection

**Figure 1:**
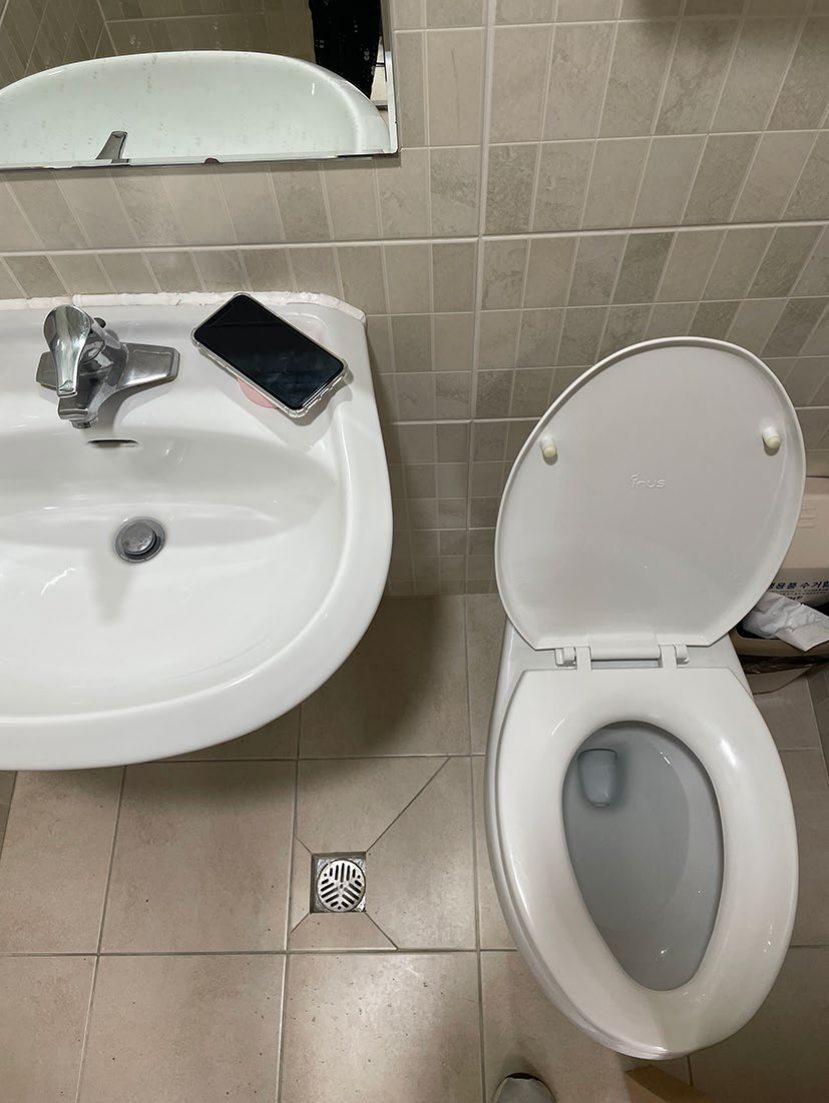
Typical bathroom settings for sound recording and the mobile application.

Urination recording was conducted using an iOS mobile application solely developed for data collection (Fig. 1). The application was installed in iPhone XR and iPhone 12 from Apple Inc., Cupertino, CA, USA.

The subject was weighed by CAS HB-150, a high resolution weight scale with a readability of 10 g and a minimum and maximum capacity of 500 g and 150 kg, respectively.

Since we did not control the amount of water intake or the time or interval of urination, which can affect the voided volume, it varied greatly even within each voiding individual, so each urination was regarded as independent.

### Voided volume prediction model and evaluation

Fivefold cross validation was performed in order to evaluate the model on all data in the dataset. An urine sound waveform is transformed into a mel-spectrogram, which is then fed as input to the 2D-CNN model for training. Also, a frequency masking method allowing masking of mel-spectrogram in the frequency domain up to 25% is applied in pre-processing to overcome overfitting due to small training set size. The output of the model is the voided volume. The mimetic diagram of voided volume estimation is demonstrated in supplementary Fig. S1.

### Statistical analysis

Paired samples t-test for equivalence was used to evaluate the statistical significance of any differences between the VV calculated based on bodyweight change after urination and the VV estimated using the iOS collection application. The equivalence of two different measurements is statistically proven if the 95% confidence interval of the mean difference is within the pre-defined equivalence margin.

To show equivalence, H0 and H1 are set as below.

- H0: |VV_pred − VV_bodyweight change|≥ δ
- H1: |VV_pred − VV_bodyweight change|< δ

As the null hypothesis (H0) has two one-sided tests (difference <  + δ or difference > − δ), 'two one-sided-tests (TOST) method' is used in equivalence testing. The p-value for this hypothesis testing as a whole is defined as the maximum p-value of two one-sided tests. If 95% CI of the difference is within the equivalence margin range (− δ, + δ), the two measurements are considered equivalent^18^. The statistical analysis and calculations were performed using the Python™ v3.6.9 programming language and its scientific computing package SciPy v1.5.4 (Python Software Foundation, Beaverton, OR, USA) and R version 4.3.1.

## Results

Total 527 subjects volunteered for this study including 300 subjects from Study 1 and 227 subjects from Study 2. After excluding 19 participants who voluntarily decided to discontinue their participation, a total of 508 subjects were enrolled in the study.

A total of 663 data points were collected from 508 enrolled subjects. After excluding 79 data points that did not meet the inclusion criteria, a total of 584 data points were included in the final analysis. Detailed description of excluded data points is summarized in Table 1.

**Table 1:** Summary of data collection.

	Study 1	Study 2	Sum
Number of bodyweight change data points collected	393	270	663
Number of data points excluded due to the lack of matching voiding sound	13	13	26
Number of data points excluded due to bodyweight change being over the capacity of the sound-based algorithm (10–1000 mL)	6	5	11
Number of data points excluded due to failure to follow instructions	17	25	42
The total number of data points included in training and test	357	227	584

**Figure 2:**
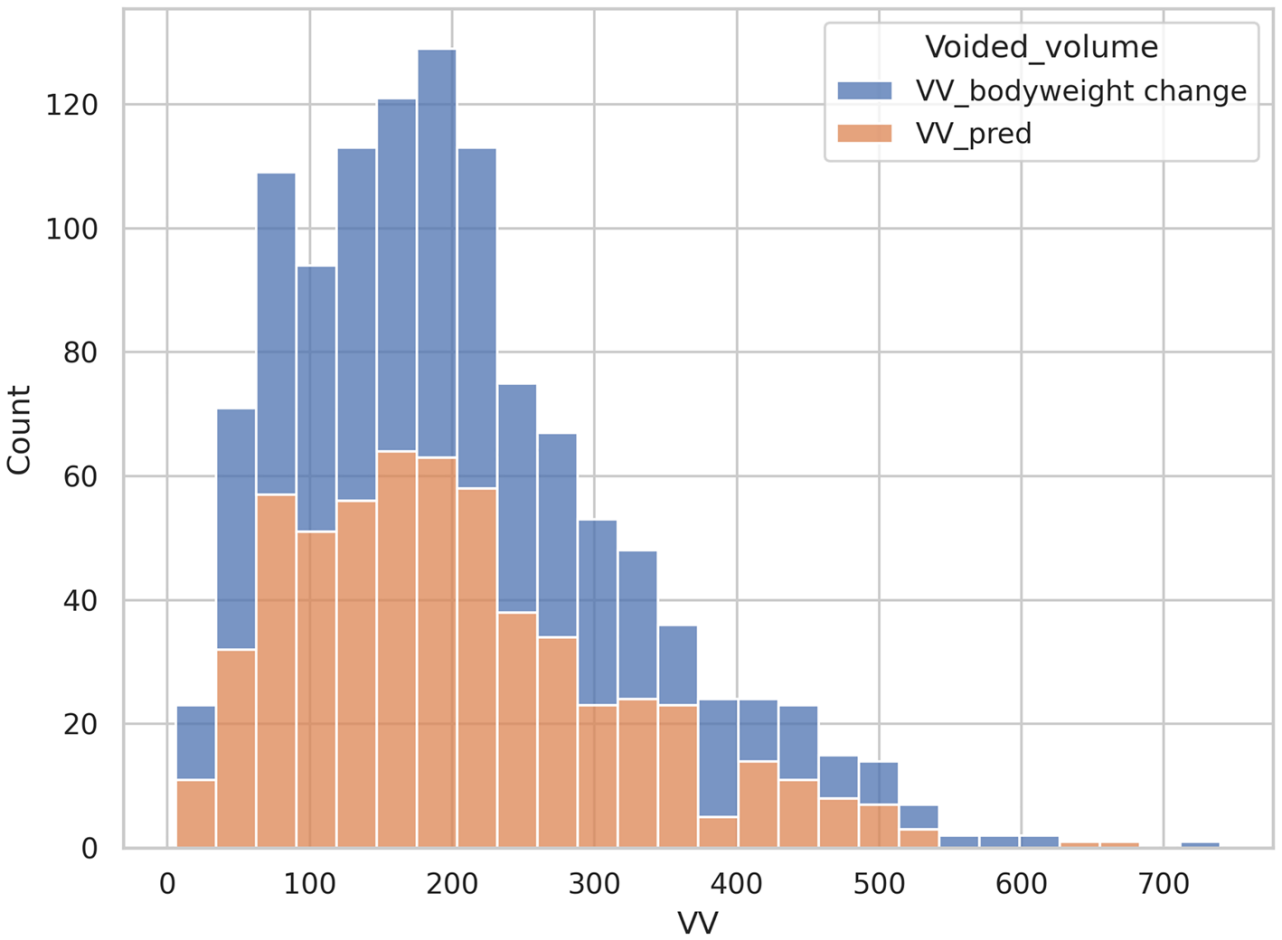
Distributions of predicted voided volume and change in weight post voiding.

**Figure 3:**
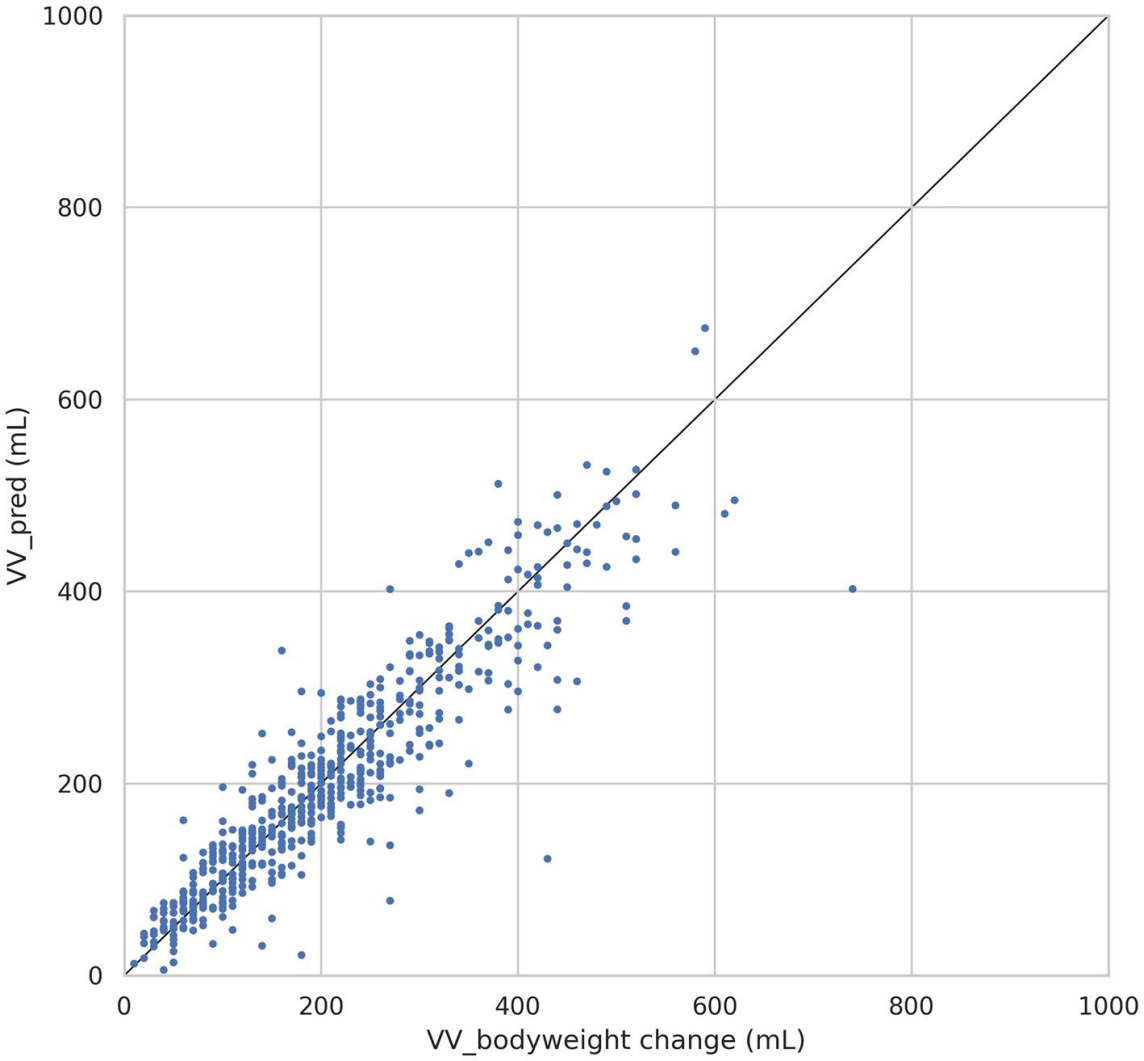
Scatter plot showing the linear correlation between estimated volume (mL) based on bodyweight changes and that by the algorithm (mL).

The mean age of the obtained data points was 60.61 (SD: ± 15.24). The mean age across the model of phone is demonstrated in Table 2. The mean VV obtained using the iOS collection application 202.6 mL (SD: ± 114.8) while the mean bodyweight change after urination was 208.0 g (SD: ± 121.5) (Table 3; Fig. 2). The statistical analysis shows strong linear correlation between the two measurements. (Pearson’s correlation coefficient = 0.92, p-value < 0.001) (Fig. 3).

**Table 2:** Summary of distribution of ages of data set.

Clinical factor	Mean age (± SD)
All (n = 584)	60.61 (± 15.24)
iPhone XR in study 1 (n = 357)	61.89 (± 12.98)
iPhone 12 in study 2 (n = 227)	58.60 (± 18.09)

**Table 3:** Summary of set.

	N	Mean (mL)	Median (mL)	Standard deviation (mL)	Standard error (mL)
VV_pred	584	202.6	184.8	114.8	4.8
VV_bodyweight change	584	208.0	190.0	121.5	5.0

*VV_pred* voided volume estimated by the algorithm, *VV_bodyweight change* voided volume estimated based on bodyweight change after urination.

**Figure 4:**
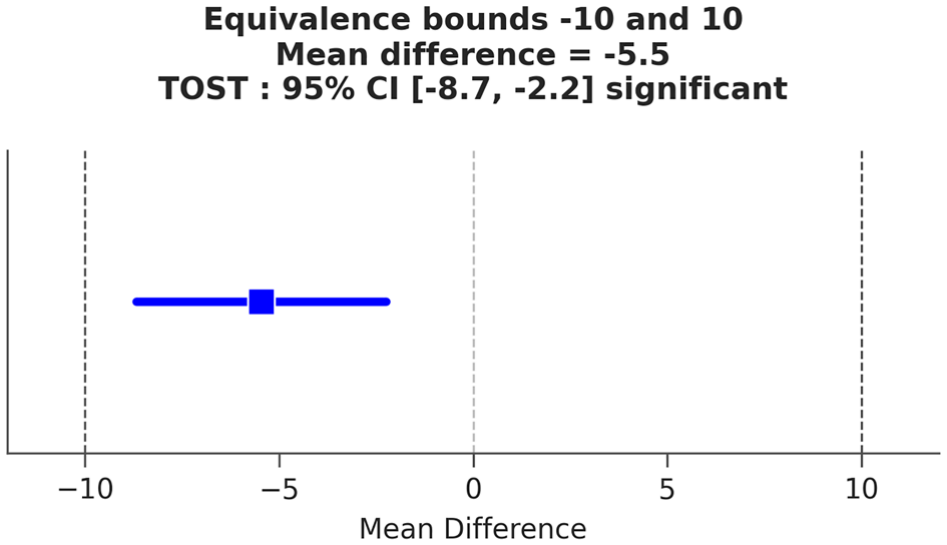
Equivalence plot from TOST results.

**Figure 5:**
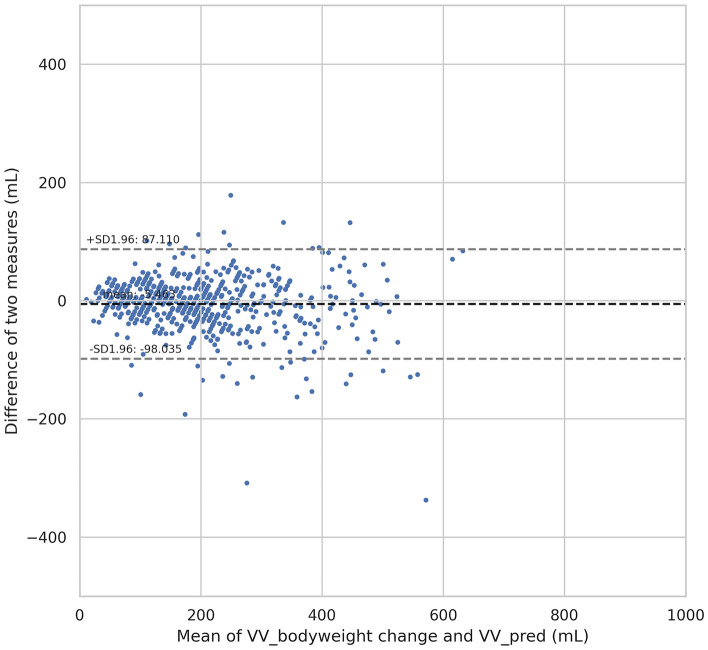
Bland–Altman plot showing difference between the estimated voided volume based on bodyweight changes and the voided volume estimated by the algorithm.

Because the scale used in this study to measure bodyweight change has a resolution of 10 g, 10 mL was set as the equivalence margin in following analyses. As shown in Fig. 4 and Table 4, the 95% CI of mean difference (− 8.8 mL, − 2.2 mL) is within the equivalence margin (− 10 mL, + 10 mL) and the maximum p-value for the TOST results (0.0103002) is smaller than 0.05. Therefore, the results demonstrate statistical equivalence between the two measurements. Additionally, we analyzed the data with a Bland–Altman plot which shows the distribution of differences between the two measurements within the Limit of Agreement (LoA). The mean difference was − 5.5 mL with LoA (− 98.0, 87.1) (Fig. 5).

**Table 4:** Summary of two one-sided-tests (TOST) results.

	t	df	p-value
t-test	− 2.79	583	0.0054
TOST lower	2.32	583	0.0103
TOST upper	− 7.91	583	< .0000001

*Df* degree of freedom.

## Discussion

The results of this study supports the use of sound-based voided volume estimation algorithm for accurately and conveniently collecting VV as a mobile voiding diary.

In this study, a Bland–Altman plot shows a mean difference of 5.5 mL with limit of agreement (− 98.0, 87.1), highly acceptable when compared to reference data. Analyses of previous studies support that this level of differences between the two measurements is highly acceptable in clinical practices. For example, C. Palnaes and P. Klarskov assessed the distribution of differences between data in a voiding diary manually recorded by patients and the actual volume of urine collected for 24 h and recorded by the nurse^19^. The Bland-Atlman plot for average urine volume during 24 h shows a limit of agreement of about 70 mL, which is similar with the results for a voided volume of each void in this study. In another study, D. R. Small et al. evaluated the measurement error of a portable bladder scanner of which use in the clinic has been well established to estimate the post-void residual. The average difference was 16.7 mL (SD: 50.2 mL), much greater than the average difference − 5.5 mL (SD: 47.2 mL) in this study^20^. The proudP’s VV estimation algorithm is based on the same AI architecture as used in this paper, but is trained on larger scale with more diverse data. Therefore, it is expected that the proudP application provides an accurate VV estimation, while significantly enhancing convenience of users by enabling mobile app-based, at-home measurements.

This study has a few limitations. First, because the sound of urination into a commercial uroflowmeter is different from the sound that the urine hits the water surface in a toilet bowl, we chose the bodyweight change before and after urination as a reference value using a high resolution weight scale instead of measuring the volume of urine directly. Accordingly, it was important to limit not to do any other actions that could affect bodyweight between before and after urination, and it needed more effort to control it and check the compliance. Second, if the assumptions made when converting urine weight to volume are different from the actual values, additional errors may occur in individual results. However, load cell and spinning disk uroflowmeters calculate VV by assuming the density of urine is approximately 1 g/ml and are already widely used in clinical practice^21^. Third, the training and performance evaluation of the AI model is based on data collected from this limited number of clinic toilets in this clinical trial. Therefore, we cannot guarantee the same performance when the measuring environments change. In terms of measurement device, although it is difficult to generalize as there are only two combinations of environment and model, there was no significant difference in mean difference and a slight difference in the LoA as shown in Supplementary Table S1 and Fig. S1. But it is difficult to distinguish the exact cause with the current data, and we want to verify it later. Finally, when the urine falls on the toilet walls or in a urinal without water, totally different sounds will be produced, and this model cannot guarantee the high accuracy for these sounds. Therefore, we limit this DL model to be used only on urination on water, and this can be easily checked by male users as they are standing in front of a toilet.

## Conclusions

The validation from this study demonstrates that the sound-based voided volume estimation algorithm provides highly accurate estimates of voided volumes when compared to the body weight change before and after urination across large population data from multi-site clinical trials. Additionally, it will enhance patient’s convenience as it eliminates the need for manual recording of voiding activities, associated potential errors, and inconvenience of carrying and using a voiding beaker. The ability to track daily voiding activities simply using a sound-based mobile app will likely improve patient compliance as well.

### Supplementary Information

Supplementary Information.

## Data Availability

The datasets analysed during the current study are available from the corresponding author on reasonable request.

## Abbreviations

TR: Training set
TE: Test set
VV: Voided volume
SD: Standard deviation
TOST: Two one-sided-tests
LoA: Limit of agreement
df: Degree of freedom
