# Supplementary material for: Validation of an algorithm for sound-based voided volume estimation
Source: Sci Rep. 2024 Jan 2;14:138. doi: 10.1038/s41598-023-50499-1 (PMC10761909; doi:10.1038/s41598-023-50499-1)
Supplement: Supplementary file 1 — Supplementary Information. [file 41598_2023_50499_MOESM1_ESM.pdf]

Legends of Supplementary Files

Supplementary Figure 1. The mimetic diagram of voided volume estimation

Supplementary Figure 2. Scatter plot of iPhone XR

Supplementary Figure 3. Bland-Altman plot of iPhone XR

Supplementary Figure 4. Scatter plot of iPhone 12

Supplementary Figure 5. Bland-Altman plot of iPhone 12

Supplementary Table 1. Summary of data across the model of phone

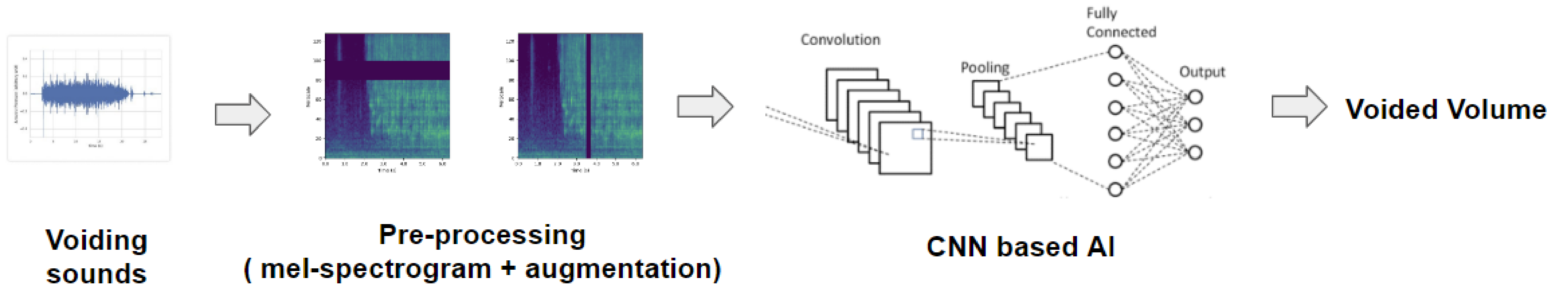
Voiding sounds
Pre-processing
(mel-spectrogram + augmentation)
Convolution
Pooling
Fully Connected
Output
CNN based AI
Voided Volume

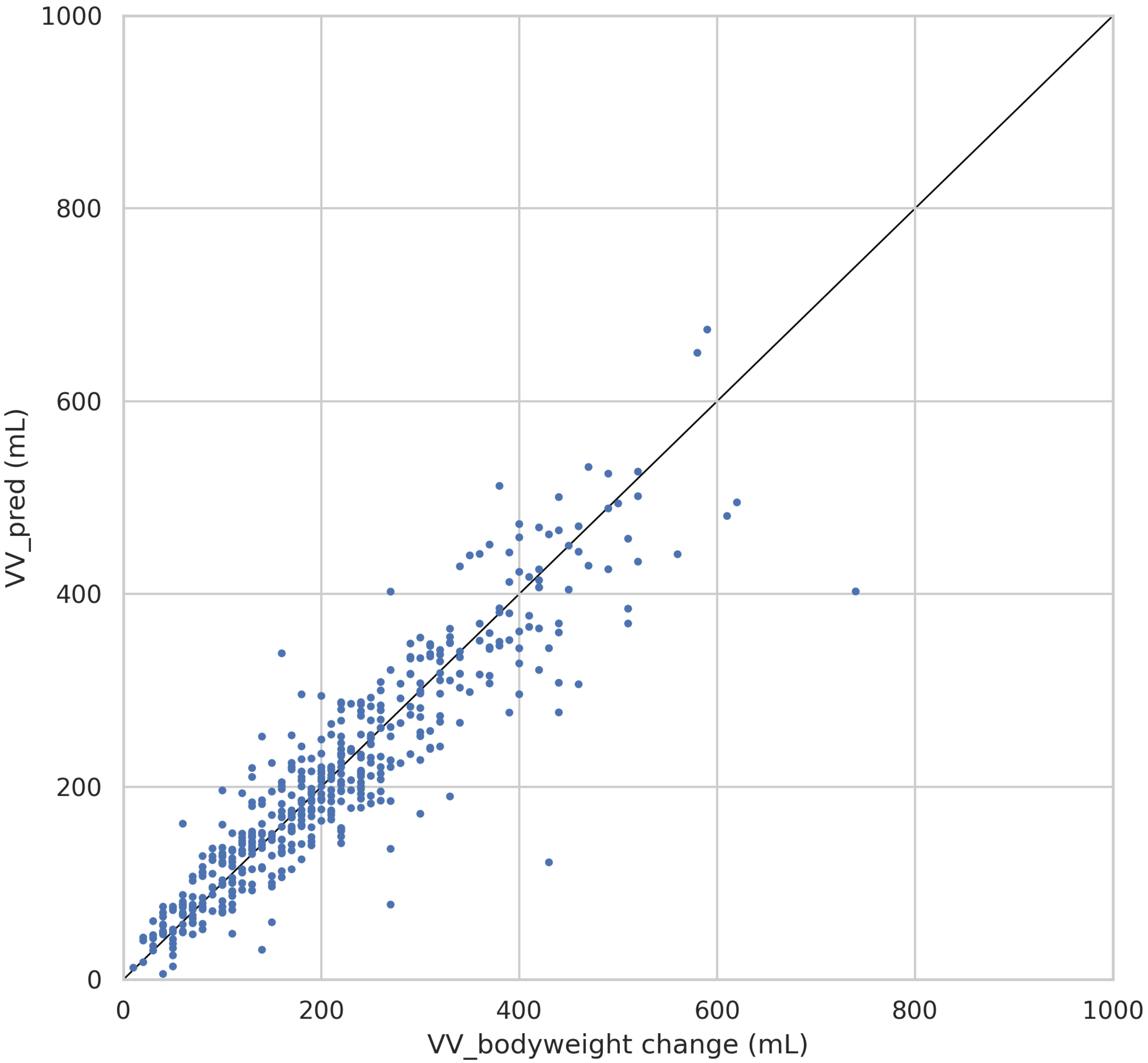
1000
800
600
400
200
0
0
200
400
600
800
1000
VV_pred (mL)
VV_bodyweight change (mL)

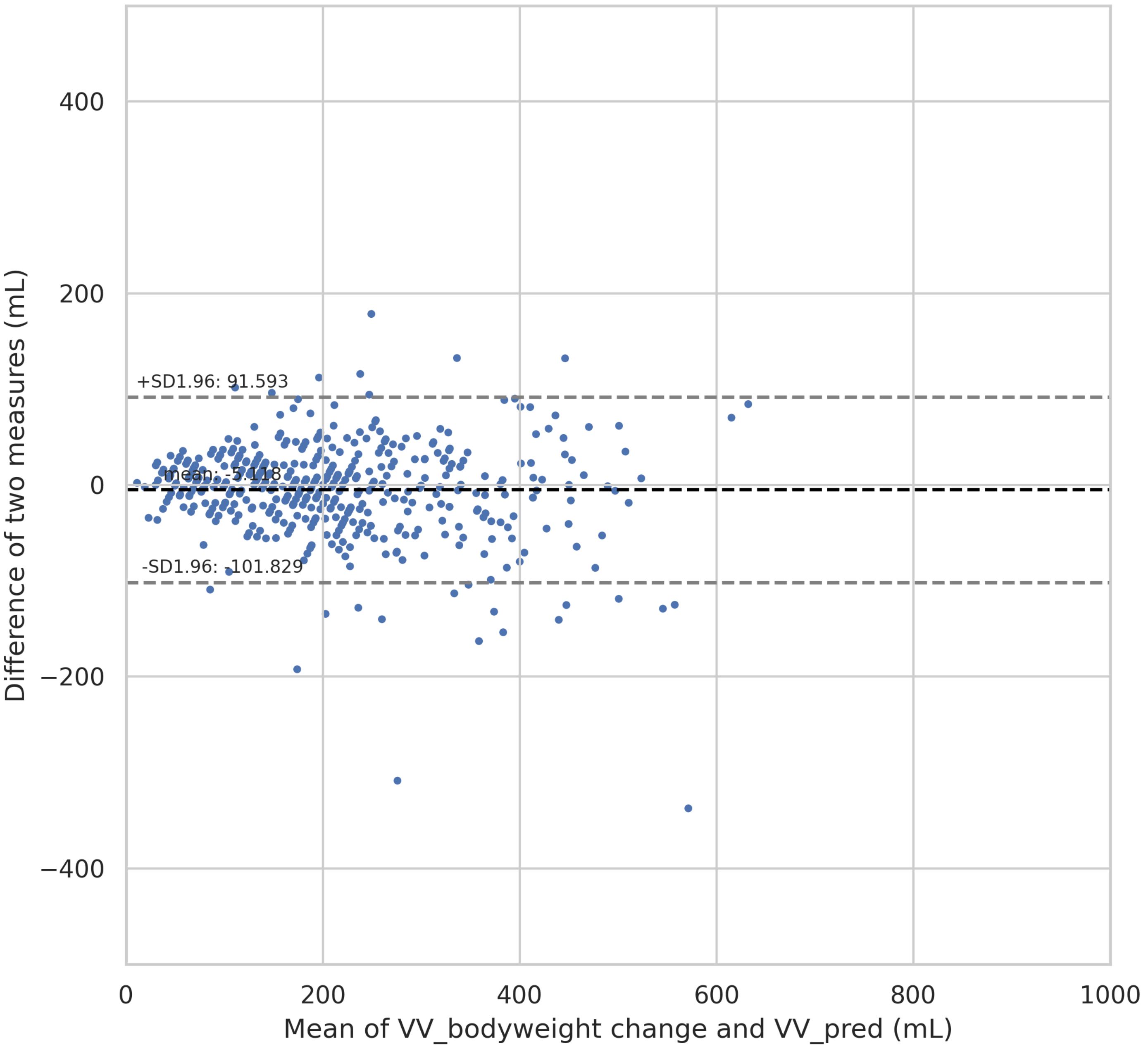

+SD1.96: 91.593
mean: -5.118
-SD1.96: -101.829
400
200
0
−200
−400
0
200
400
600
800
1000
Difference of two measures (mL)
Mean of VV_bodyweight change and VV_pred (mL)

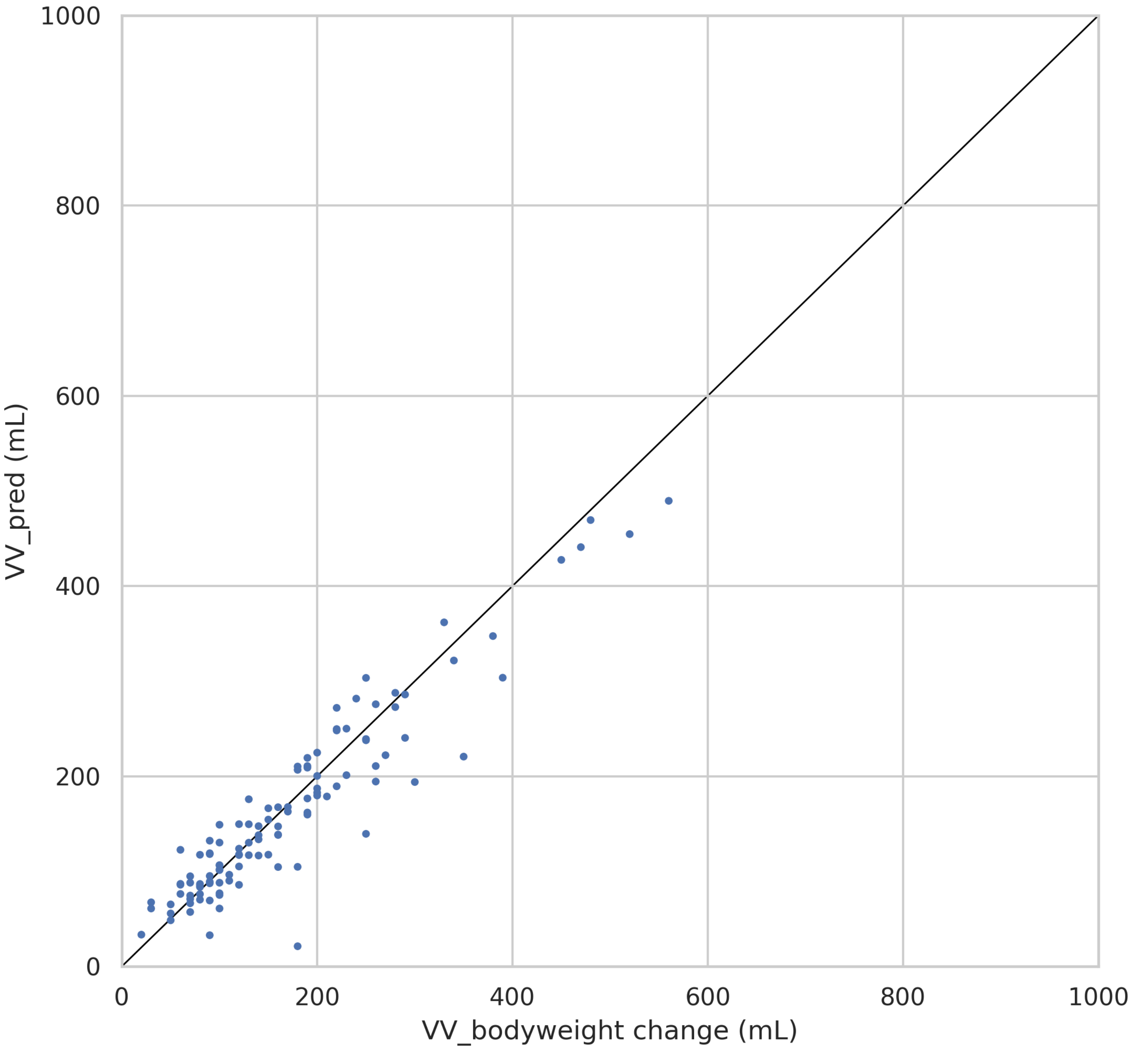

1000
800
600
400
200
0
0
200
400
600
800
1000
VV_bodyweight change (mL)
VV_pred (mL)

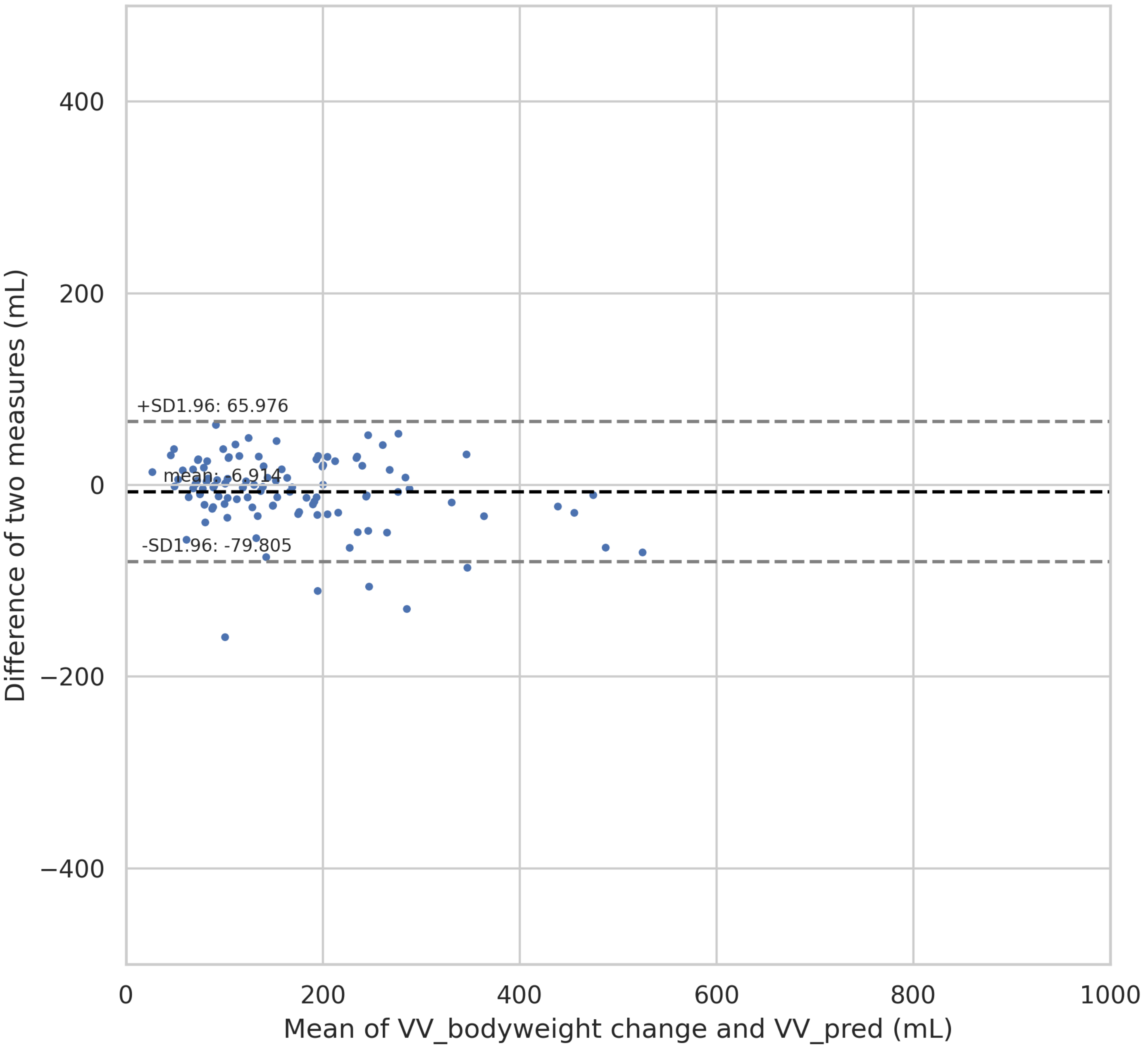

+SD1.96: 65.976
mean: -6.914
-SD1.96: -79.805
400
200
0
−200
−400
0
200
400
600
800
1000
Difference of two measures (mL)
Mean of VV_bodyweight change and VV_pred (mL)

Supplementary Table 1. Summary of data across the model of phone

	Number of data	Body weight change after urination (mean+-sd)	VV obtained using the iOS collection application (mean+-sd)	Pearson's correlation coefficient (p-value)	The mean difference (LoA)
**iPhone XR**	472	216.9 (123.1)	211.1 (116.7)	0.92 (2.1e-189)	-5.1 (-101.8, 91.6)
**iPhone 12**	112	170.6 (107.2)	163.7 (97.5)	0.94 (1.5e-52)	-6.9 (-79.8, 66.0)
